# Integrated Inflammatory Biomarker Profiling Differentiates Degrees of Body Mass Index Beyond Intestinal Barrier-Related Markers

**DOI:** 10.3390/cells15090763

**Published:** 2026-04-24

**Authors:** Theocharis Koufakis, Areti Kourti, Katerina Thsiadou, Paraskevi Karalazou, Ioannis Georgiadis, Dimitrios Patoulias, Djordje S. Popovic, Giuseppe Maltese, Alexander Kokkinos, Kalliopi Kotsa, Michael Doumas, Carel W. le Roux, Kali Makedou

**Affiliations:** 1 Second Propaedeutic Department of Internal Medicine, Aristotle University of Thessaloniki, Hippokration General Hospital, 54642 Thessaloniki, Greece; 2 Laboratory of Biochemistry, School of Medicine, Aristotle University of Thessaloniki, AHEPA University Hospital, 54636 Thessaloniki, Greece; 3 Clinic for Endocrinology, Diabetes and Metabolic Disorders, Clinical Centre of Vojvodina, Medical Faculty, University of Novi Sad, 21000 Novi Sad, Serbia; 4 Unit for Metabolic Medicine, Cardiovascular Division, Faculty of Life Sciences & Medicine, King’s College, London WC2R 2LS, UK; 5 First Department of Propaedeutic Internal Medicine and Diabetes Center, Medical School, National and Kapodistrian University of Athens, Laiko General Hospital, 11527 Athens, Greece; 6 Division of Endocrinology and Metabolism, First Department of Internal Medicine, Medical School, Aristotle University of Thessaloniki, AHEPA University Hospital, 54636 Thessaloniki, Greece; 7 School of Medicine, University College Dublin, DO4 V1W8 Dublin, Ireland

**Keywords:** obesity, overweight, systemic inflammation, biomarkers, composite index, gut barrier

## Abstract

**Highlights:**

**What are the main findings?**

- A composite inflammatory biomarker index differentiates obesity classes, whereas individual biomarkers show limited discriminatory capacity.
- Intestinal barrier-related biomarkers, including β-defensin-2 and REG3α, do not distinguish between obesity classes in people without diabetes.

**What are the implication of the main findings?**

- Integrated biomarker approaches may provide a more sensitive representation of obesity-related biological burden than single markers.
- Composite inflammatory indices may support improved phenotyping and risk stratification across obesity classes.

**Abstract:**

Obesity is characterized by low-grade systemic inflammation and alterations in gut-related immune pathways that may contribute to metabolic dysfunction. Composite biomarker indices may better capture these complex processes than individual markers, although their performance may differ across biological domains. In this cross-sectional study, 88 adults without diabetes or infection were categorized as BMI < 25 kg/m^2^ (*n* = 20), BMI 25–29.9 kg/m^2^ (*n* = 34), or BMI ≥ 30 kg/m^2^ (*n* = 34). Circulating biomarkers reflecting systemic inflammation (high-sensitivity C-reactive protein, ferritin, interleukin-6, presepsin) and intestinal barrier-related activity (β-defensin-2, regenerating islet-derived protein 3 alpha) were measured and subsequently combined into two composite indices: the Inflammatory Load Index, derived from inflammatory markers, and the Barrier Activation Index, derived from barrier-related markers. Group differences were assessed using analysis of variance with post hoc testing. Additional analyses included effect size estimation, receiver operating characteristic (ROC) analysis, and logistic regression. Individual biomarkers showed limited differences across BMI categories. The Inflammatory Load Index differed significantly across BMI categories (*p* = 0.040), with higher values observed in individuals with BMI ≥ 30 kg/m^2^ compared with those with BMI 25–29.9 kg/m^2^ (*p* = 0.032; Cohen’s d = 0.80), while the Barrier Activation Index did not differ (*p* = 0.257). In ROC analysis, the Inflammatory Load Index discriminated BMI ≥ 30 kg/m^2^ with an area under the curve of 0.720 (95% confidence interval 0.576–0.851), yielding 77.8% sensitivity and 67.7% specificity. Each one standard deviation increase in the index was associated with higher odds of obesity (odds ratio 2.34, 95% confidence interval 1.22–4.49; *p* = 0.011). In conclusion, a composite inflammatory biomarker index, but not a barrier-related index, differentiates degrees of BMI in individuals without diabetes. These findings support integrated biomarker approaches for reflecting obesity-related biological burden beyond single markers. However, these observations are based on cross-sectional data and do not imply causality.

## 1. Introduction

Obesity is a chronic disease characterized by persistent, low-grade systemic inflammation, reflecting a complex interplay between metabolic and immune processes [1]. Excess adiposity leads to structural and functional alterations in adipose tissue, promoting immune cell infiltration and activation, which in turn contribute to a state of sustained inflammatory signaling [2]. Importantly, the relationship between obesity and inflammation is bidirectional. While increased fat mass initiates and amplifies inflammatory responses, chronic inflammation itself can further impair metabolic homeostasis, favor fat redistribution to ectopic depots, and promote the development of insulin resistance [3]. This reciprocal interaction creates a self-perpetuating cycle that underlies the progression from excess adipocyte mass to obesity-related complications, including type 2 diabetes and cardiovascular disease [4].

In parallel, accumulating evidence highlights the gut microbiota as a key regulator of metabolic homeostasis and a potential contributor to obesity-related inflammation [5]. Alterations in the composition and functional capacity of the intestinal microbiome, commonly referred to as gut dysbiosis, have been consistently associated with excess adiposity and adverse metabolic profiles [6]. Dysbiosis may influence host metabolism through multiple mechanisms, including enhanced energy harvest from the diet, the modulation of intestinal barrier integrity, and interaction with host immune pathways [7]. Impairment of the gut barrier may facilitate the translocation of luminal components into the circulation, thereby promoting systemic inflammatory responses [8]. Notably, this interaction appears to be reciprocal, as obesity-related metabolic disturbances can further reshape the gut microbial ecosystem, reinforcing a cycle that links dysbiosis, inflammation, and metabolic dysfunction [9].

A range of circulating biomarkers reflect the interaction between systemic inflammation, innate immune activation, and intestinal barrier-related responses in metabolic disease. High-sensitivity C-reactive protein (hs-CRP) and interleukin-6 (IL-6) are established indicators of low-grade systemic inflammation and have been consistently associated with insulin resistance and adverse cardiometabolic risk profiles [10]. Ferritin, beyond its role in iron storage, also behaves as an acute-phase reactant and has been linked to metabolic dysfunction and hepatic involvement [11]. Presepsin, a marker of monocyte and macrophage activation, reflects innate immune system engagement, chronic inflammation, and cardiometabolic stress in people with infection [12]. In parallel, β-defensin-2 is an antimicrobial peptide involved in mucosal defense, while regenerating islet-derived protein 3 alpha (REG3α) is associated with intestinal barrier integrity and host–microbiota interactions [13]. Collectively, these biomarkers provide complementary information on immune and barrier-related pathways that are integral to the development and progression of metabolic disorders [14].

Despite growing recognition of the complex interplay between inflammation, innate immunity, and gut barrier function in obesity, there is still a need for biomarkers that can meaningfully capture these complex pathophysiological processes and discriminate between different metabolic phenotypes [15]. Single biomarkers often provide only a partial view of this multifaceted biology, whereas composite indices integrating multiple pathways may better reflect the overall biological burden [16]. Such integrated scores may offer improved sensitivity in identifying subtle differences across adiposity states. While composite biomarker approaches have been explored in several disease settings, their domain-specific performance in obesity—particularly when directly comparing inflammatory and intestinal barrier-related pathways within the same population—remains insufficiently characterized. In addition, it is unclear whether such integrated indices retain discriminatory capacity in individuals without overt metabolic disease, where biological differences are expected to be more subtle. The present study aimed not only to evaluate the behavior of composite inflammation- and barrier-related biomarker indices across body mass index (BMI) categories, but also to compare their relative ability to capture adiposity-related biological variation within a relatively uniform metabolic background.

## 2. Methods

### 2.1. Study Population

Participants were consecutively enrolled between January and May 2024 from the Obesity Clinics of Hippokration General Hospital and AHEPA University Hospital in Thessaloniki, Greece. Adults (≥18 years) were classified by BMI as normal-weight individuals (BMI 18.5–24.9 kg/m^2^), overweight (25.0–29.9 kg/m^2^) or obese (≥30 kg/m^2^) [17]. Inclusion required a stable body weight over the preceding three months and the absence of acute clinical conditions at the time of assessment. Individuals were excluded if they had diabetes, had an established cardiovascular disease, were active smokers, or had autoimmune or malignant disorders, significant renal impairment (estimated glomerular filtration rate < 60 mL/min/1.73 m^2^), or liver dysfunction (defined by known disease or aminotransferase levels > 2× the upper limit of normal). Additional reasons for exclusion included recent surgery, acute inflammatory conditions, or treatment with corticosteroids, immunosuppressive agents, and anti-obesity drugs. Former smokers were considered eligible if abstinence exceeded six months.

### 2.2. Clinical Evaluation and Laboratory Measurements

Participants underwent standardized clinical evaluation, including anthropometric assessment and collection of blood samples under fasting conditions. Body weight and height were measured using calibrated instruments, and BMI was calculated. Following overnight fasting, venous blood samples were collected, promptly processed, and stored at −20 °C until analysis.

Circulating biomarkers were measured using validated immunoassay-based methodologies. Ferritin and IL-6 were measured using electrochemiluminescence-based methods, while hs-CRP was assessed via an automated immunoturbidimetric approach. Presepsin concentrations were determined using a sandwich-format enzyme immunoassay (FineTest, Wuhan, China) with an analytical range spanning low nanogram levels and acceptable intra- and inter-assay precision (approximately 5%). β-defensin-2 and REG3α were similarly measured using sandwich immunoassays (FineTest, Wuhan, China) with picogram-level detection ranges and comparable analytical variability. All measurements were conducted in a single certified laboratory using harmonized procedures to limit analytical variability and maintain measurement consistency.

### 2.3. Statistical Analysis

Continuous variables were presented as mean ± standard deviation (SD), and categorical variables as counts and percentages. Distributional assumptions were assessed using the Shapiro–Wilk test. Differences across BMI categories were examined using one-way analysis of variance for normally distributed variables, followed by Holm-adjusted pairwise comparisons when the overall test was significant. For non-normally distributed variables, the Kruskal–Wallis test was applied.

Composite indices were constructed after log transformation and standardization of biomarker values. The Inflammatory Load Index was defined as the mean of standardized hs-CRP, ferritin, IL-6, and presepsin values, whereas the Barrier Activation Index was calculated as the mean of standardized β-defensin-2 and REG3α values. Because these indices were derived from z-scores, the resulting standardized scores are centered at zero, where positive values denote relatively greater and negative values relatively lower biomarker burden within the cohort. Effect sizes for pairwise comparisons were estimated using Cohen’s d. All biomarkers contributed equally to the composite index following standardization. This approach was selected to ensure comparability across markers, preserve interpretability, and minimize the risk of overfitting given the sample size, in the absence of prior evidence supporting differential weighting. Alternative data-driven approaches for weighting, such as principal component analysis or other dimensionality reduction techniques, may provide additional insight into biomarker structure and relative contributions, but were not applied in the present study due to the modest sample size and exploratory design.

Discriminative performance across BMI categories was assessed using receiver operating characteristic (ROC) curve analysis, with estimation of the area under the curve (AUC) and corresponding 95% confidence intervals. The optimal cut-off point was identified based on the Youden index [18]. Logistic regression was used to assess the association between the Inflammatory Load Index and obesity (compared with overweight), with results expressed per one SD increase. Sensitivity analyses were conducted to assess the robustness of the composite index. A leave-one-out approach was applied, whereby the Inflammatory Load Index was recalculated after sequential exclusion of each individual biomarker, and the corresponding between-group comparisons were repeated. These analyses were intended to assess the internal robustness of the composite index rather than to serve as a formal validation procedure. All analyses were conducted using Python (version 3.11) with standard scientific libraries (pandas, NumPy, SciPy, scikit-learn). A two-sided *p*-value < 0.05 was considered statistically significant. Formal power calculations were not performed, as the study was designed as an exploratory cross-sectional analysis without a prespecified primary outcome. In such settings, post hoc power estimation is of limited interpretative value and is not routinely recommended [19]. Instead, emphasis was placed on reporting effect sizes and confidence intervals to provide a more informative assessment of the magnitude and precision of the observed associations.

### 2.4. Ethical Considerations

The study protocol was approved by the bioethics committee of the Aristotle University of Thessaloniki (approval no. 6.233/29.7.2020). Written informed consent was obtained from every participant prior to enrolment.

## 3. Results

### 3.1. Primary Analysis

A total of 88 adults were included and categorized into normal weight (*n* = 20), overweight (*n* = 34), and obesity (*n* = 34) groups. Age did not differ significantly between groups (*p* = 0.163), whereas BMI increased as expected across categories (22.51 ± 1.29, 27.25 ± 1.22, and 34.32 ± 3.83 kg/m^2^, respectively; *p* < 0.01). Among individual biomarkers, presepsin differed significantly across BMI categories (*p* = 0.008). In contrast, no statistically significant differences were observed for hs-CRP (*p* = 0.144), ferritin (*p* = 0.199), or IL-6 (*p* = 0.126). Similarly, biomarkers reflecting barrier-related activity did not differ across BMI categories, including β-defensin-2 (*p* = 0.976) and REG3α (*p* = 0.226). Baseline demographic, anthropometric, and biomarker characteristics across BMI categories are presented in Table 1.

**Figure 1:**
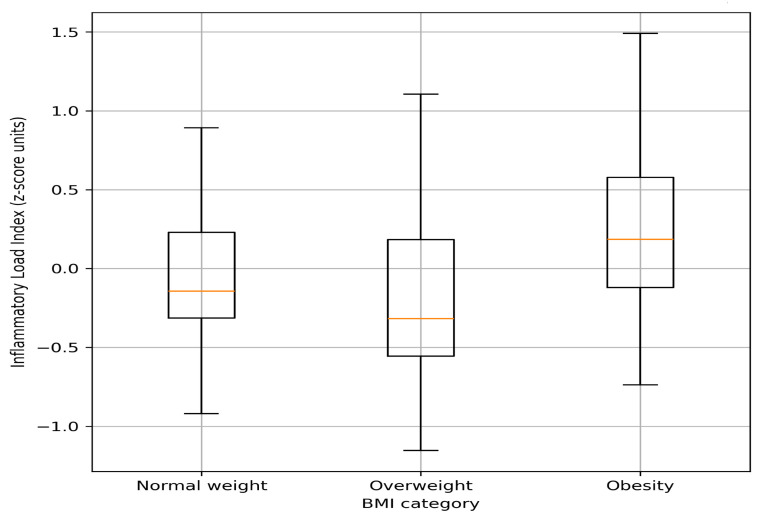
Distribution of the Inflammatory Load Index across body mass index (BMI) categories. Boxes represent interquartile ranges, horizontal orange lines indicate medians, and whiskers denote the range of the data.

In contrast, the Inflammatory Load Index differed significantly across BMI categories (mean ± SD: normal weight −0.06 ± 0.44, overweight −0.18 ± 0.53, obesity 0.22 ± 0.54; *p* = 0.040, Figure 1). Post hoc analysis demonstrated that individuals with BMI ≥ 30 kg/m^2^ obesity had significantly higher values compared with those with BMI 25–29.9 kg/m^2^ (mean difference 0.45 SD units; Holm-adjusted *p* = 0.032), whereas no significant differences were observed between BMI < 25 kg/m^2^ and BMI 25–29.9 kg/m^2^ or between BMI < 25 kg/m^2^ and BMI ≥ 30 kg/m^2^.

The Barrier Activation Index did not differ significantly across BMI categories (BMI < 25 kg/m^2^ −0.11 ± 1.23, BMI 25–29.9 kg/m^2^ 0.09 ± 0.65, and BMI ≥ 30 kg/m^2^ −0.04 ± 0.61; *p* = 0.257), indicating that intestinal barrier-related biomarkers did not discriminate between obesity classes in this cohort.

### 3.2. Secondary Analyses

To further characterize the magnitude of group differences, effect size estimation was performed (Table 2). The BMI 25–29.9 kg/m^2^ versus and BMI ≥ 30 kg/m^2^ comparison yielded a Cohen’s d of 0.80, indicating a moderate-to-large effect size. In contrast, comparisons involving BMI < 25 kg/m^2^ were associated with smaller effect sizes and did not reach statistical significance.

Sensitivity analyses using a leave-one-out approach demonstrated that the overall pattern of results remained unchanged after sequential exclusion of each individual biomarker from the Inflammatory Load Index. Across all iterations, values remained higher in individuals with obesity compared with those with overweight. The magnitude of the between-group differences and corresponding effect sizes were only minimally affected, and statistical significance was preserved, indicating that the discriminatory performance of the composite index was not driven by any single biomarker.

Non-parametric analysis confirmed the robustness of the findings. The Inflammatory Load Index remained different across BMI categories when assessed using the Kruskal–Wallis test (*p* = 0.011), indicating that the observed differences were not dependent on distributional assumptions and supporting the stability of the results.

**Figure 2:**
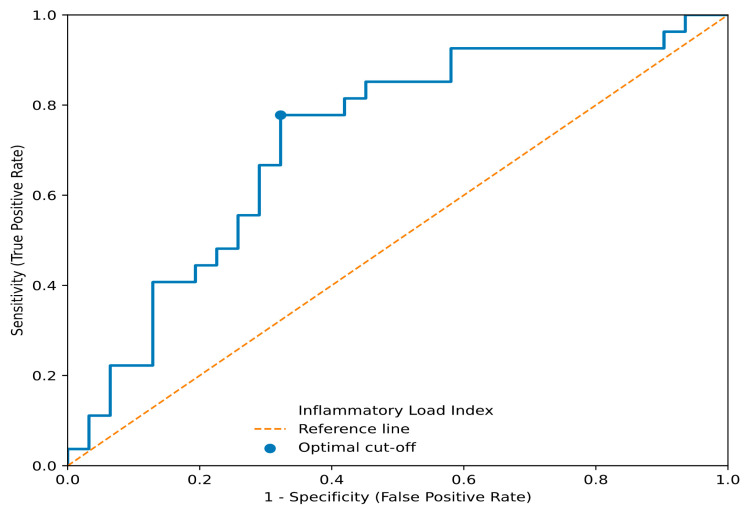
Receiver operating characteristic curve of the Inflammatory Load Index for discrimination of obesity versus overweight.

ROC analysis demonstrated that the Inflammatory Load Index discriminated individuals with BMI ≥ 30 kg/m^2^ from those with and BMI 25–29.9 kg/m^2^ with an AUC of 0.720 [95% confidence interval (CI) 0.576–0.851] (Figure 2). At the optimal threshold, sensitivity was 77.8% and specificity was 67.7%, indicating moderate but imprecisely estimated discriminatory performance. These findings further support the ability of the composite index to discriminate between obesity classes beyond conventional single biomarkers.

In logistic regression analysis, each one SD increase in the Inflammatory Load Index was associated with 2.34-fold higher odds of BMI ≥ 30 kg/m^2^ compared with BMI 25–29.9 kg/m^2^ (95% CI 1.22–4.49; *p* = 0.011). The composite Inflammatory Load Index demonstrated superior discriminatory performance compared with its individual components (Table 3), all of which showed lower ability to differentiate between BMI ≥ 30 kg/m^2^ and BMI 25–29.9 kg/m^2^. In contrast, neither individual barrier-related biomarkers nor the composite Barrier Activation Index exhibited meaningful discriminatory capacity. This finding should be interpreted with caution, as the more limited number of biomarkers included in this index may not fully capture the complexity of intestinal barrier-related processes. In addition, currently available circulating markers of barrier function are less well established compared with inflammatory biomarkers, which may further contribute to the observed differences.

## 4. Discussion

In this cross-sectional study of adults without diabetes, we demonstrate that a composite inflammatory biomarker index can discriminate between obesity classes, even within a relatively uniform metabolic background. These findings should be considered exploratory and hypothesis-generating, given the sample size and the variability observed in individual biomarkers. Specifically, the Inflammatory Load Index was higher in individuals with BMI ≥ 30 kg/m^2^ compared with BMI 25–29.9 kg/m^2^, with a moderate-to-large effect size and consistent findings across multiple analytical approaches, including ROC analysis and logistic regression. In contrast, individual inflammatory biomarkers showed limited discriminatory ability, and neither individual nor composite barrier-related markers differed across BMI categories. These findings extend existing knowledge by demonstrating that the value of biomarker integration in obesity is domain-dependent, with inflammatory pathways showing consistent discriminatory capacity, whereas intestinal barrier-related pathways do not exhibit similar behavior within the same analytical framework [20]. The ability of the index to differentiate between BMI ≥ 30 kg/m^2^ and BMI 25–29.9 kg/m^2^ in people without diabetes supports its ability to capture cross-sectional differences across BMI categories beyond overt disease states, representing a novel and clinically relevant approach in obesity research that warrants further validation.

Importantly, the present study goes beyond confirming the general principle that combining biomarkers improves signal detection. By directly contrasting two biologically distinct domains within the same population, our findings indicate that the discriminatory performance of composite indices is not uniform across pathophysiological pathways. While inflammatory integration captured gradations of adiposity even in individuals without diabetes, barrier-related markers did not demonstrate comparable behavior, either individually or as a composite index. This suggests that pathway relevance, rather than statistical aggregation alone, is a critical determinant of biomarker utility in obesity phenotyping.

An important observation of the present study is that the composite inflammatory index did not significantly differentiate individuals with normal weight from those overweight. This finding suggests that the relationship between adiposity and systemic inflammation may not be linear across the full BMI spectrum. Rather, inflammatory activation may become more pronounced beyond a threshold of adiposity, with more detectable biological differences emerging between overweight and obesity. In a metabolically homogeneous population without diabetes, early or subtle inflammatory changes may remain below the detection capacity of circulating biomarkers, even when combined into composite indices.

The superior performance of the composite Inflammatory Load Index compared with individual biomarkers likely reflects the multifactorial nature of obesity-related inflammation. Excess adiposity is associated with a complex network of interconnected pathways involving immune activation, metabolic stress, and tissue remodeling, none of which can be fully captured by a single circulating marker [21]. Individual biomarkers are often subject to substantial biological variability and may reflect only specific aspects of this process, thereby limiting their discriminatory capacity. In contrast, integrating multiple biomarkers into a composite index provides a more integrated representation of the inflammatory milieu, reducing noise and enhancing signal detection [22,23]. This interpretation is further supported by the leave-one-out sensitivity analyses, in which the discriminatory performance of the index remained consistent after sequential exclusion of each component biomarker, suggesting that the observed signal reflects the integrated contribution of multiple markers rather than the influence of a single dominant component. While these observations support the internal stability of the composite index, the absence of independent validation should be taken into account when interpreting its performance, as estimates derived within a single dataset may be susceptible to overfitting and overestimation of discriminatory ability. These findings also suggest that the clinical utility of composite biomarker strategies in obesity depends not only on integration itself, but also on the selection of biologically informative pathways, with inflammatory processes appearing to provide a more sensitive signal of adiposity-related burden in early or intermediate stages.

Conversely, the absence of discriminatory capacity for barrier-related biomarkers warrants careful consideration. Intestinal barrier dysfunction is increasingly recognized as a contributor to metabolic disease; however, its relationship with adiposity may not be linear or uniformly expressed across individuals. Rather than reflecting a gradual, dose-dependent process, barrier activation may occur in a threshold-dependent manner or be restricted to specific metabolic phenotypes characterized by more advanced or dysregulated states [24]. Additionally, circulating markers of barrier-related immune activity may be influenced by various factors beyond adiposity, including dietary patterns, microbiota composition, and host–environment interactions, potentially attenuating their association with BMI [25]. In this context, the lack of differences observed in the present study suggests that barrier-related pathways may be less sensitive indicators of adiposity per se, particularly in individuals without diabetes, and may instead reflect more advanced stages or distinct trajectories of metabolic dysfunction. This interpretation likely reflects a combination of threshold-dependent biology and the limited sensitivity of the selected circulating markers to detect early or subtle barrier-related alterations. However, our findings should not be interpreted as definitive evidence against a role for intestinal barrier dysfunction in obesity. Rather, they likely reflect limitations related to the selected circulating biomarkers and their ability to capture the complexity and dynamics of intestinal barrier function. Alternative or more comprehensive approaches, including functional assessments or broader biomarker panels, may provide improved sensitivity in detecting barrier-related alterations.

An important aspect of the present study is the inclusion of people without diabetes, which allows for a more refined assessment of adiposity-related biological differences independent of overt hyperglycemia. By minimizing the confounding effects of hyperglycemia and advanced metabolic dysregulation, this approach provides a more homogeneous metabolic background against which the impact of increasing adiposity can be evaluated. Notably, despite this relative metabolic uniformity, the composite inflammatory index was still able to differentiate between BMI ≥ 30 kg/m^2^ and BMI 25–29.9 kg/m^2^, suggesting that it reflects differences in adiposity-related biological profiles beyond glucose metabolism. However, the cross-sectional nature of the study does not allow conclusions to be reached regarding the temporal sequence or progression of these changes [26,27].

From a clinical perspective, these findings suggest that such composite indices may have potential utility as adjunctive tools for risk stratification, particularly in identifying individuals with relatively higher inflammatory profiles within similar BMI categories. In practical terms, such an index may be most relevant in settings where individuals with comparable BMI exhibit heterogeneous metabolic risk profiles, enabling identification of those with relatively higher inflammatory burden who may warrant closer monitoring or earlier therapeutic intervention despite similar anthropometric status. This is consistent with evidence from other chronic complex diseases, such as rheumatoid arthritis and systemic lupus erythematosus, where multi-biomarker panels integrating cytokines and related pathways have demonstrated improved accuracy in assessing disease activity and guiding therapeutic decisions compared with individual markers [28]. This may be particularly relevant in the context of personalized medicine, where improved phenotyping of individuals with obesity could inform targeted interventions [29]. The moderate discriminatory performance of the composite inflammatory index suggests potential utility for obesity-related phenotyping; however, it remains insufficient for standalone clinical application. As such, its role may be better suited to research settings or as an adjunctive tool for biological stratification, pending further validation in larger and more diverse populations. Our findings also suggest that the utility of multi-biomarker approaches may be stage-dependent, with greater discriminatory capacity in more advanced adiposity states. However, further studies are required to validate these observations, assess longitudinal associations with clinical outcomes, and determine whether such indices provide incremental value beyond established clinical and biochemical parameters.

The present study has several strengths that support the validity and relevance of its findings. First, the use of a composite biomarker approach integrating multiple inflammatory pathways provides a more comprehensive assessment of obesity-related biological processes compared with single-marker analyses. Second, the inclusion of well-characterized, people without diabetes allowed for the evaluation of adiposity-related differences within a metabolically comparable context, minimizing the confounding influence of overt glycemic disease. Third, the application of multiple complementary analytical approaches, including effect size estimation, ROC analysis, and logistic regression, enhances the robustness and interpretability of the results. Finally, the simultaneous assessment of both inflammatory and barrier-related biomarkers enabled a comparative evaluation of distinct pathophysiological domains, offering additional insight into their relative contribution to adiposity-related metabolic alterations.

However, several limitations should be acknowledged. The cross-sectional design precludes conclusions regarding causality or temporal relationships between adiposity and biomarker profiles. The sample size, although adequate to detect differences in the composite inflammatory index, was relatively modest and may have limited the ability to identify more subtle associations, particularly for barrier-related biomarkers. In addition, the relatively wide confidence intervals observed in some analyses, including ROC curves, indicate a degree of imprecision in effect estimates that should be taken into account when interpreting the findings. In addition, the composite index was derived and evaluated within the same dataset, and no external validation or cross-validation was performed, which may have resulted in optimistic estimates of discriminatory performance. Another limitation relates to the unequal representation of biomarker domains in the composite indices. The Barrier Activation Index was constructed from fewer biomarkers than the Inflammatory Load Index, which may have constrained its ability to capture the complexity of intestinal barrier-related pathways and potentially attenuated its discriminatory performance. Importantly, the two indices were designed to reflect distinct biological domains rather than to serve as directly comparable models, and differences in performance should be interpreted in this context. Accordingly, the observed differences between inflammatory and barrier-related indices should be interpreted with caution, as they may partially reflect differences in biomarker panel composition rather than exclusively true biological differences between these domains. Therefore, the present findings should not be interpreted as a head-to-head comparison of the methodological performance of the two indices.

We did not evaluate incremental predictive value beyond established clinical or anthropometric measures. Such analyses were not feasible given the cross-sectional design, the absence of independent clinical predictors such as waist circumference, and the use of BMI to define outcome categories, which precludes its use as an independent comparator due to circularity. In addition, the study population consisted exclusively of diabetes-free individuals, which, while reducing metabolic heterogeneity, may limit the generalizability of the findings to populations with established diabetes or more advanced metabolic disease. We did not account for potential confounding factors such as dietary habits, physical activity, socioeconomic status, or gut microbiome composition, all of which may influence inflammatory and barrier-related biomarker profiles. Although sensitivity analyses were performed, the index was constructed using equal weighting of biomarkers without data-driven optimization and may not fully reflect the relative contribution of individual components. Biomarker measurements were performed at a single time point, and intra-individual variability cannot be excluded. Finally, external validation in independent cohorts is required to confirm the reproducibility and broader applicability of the composite indices.

In conclusion, a composite inflammatory biomarker index, but not a barrier-related index, appears to differentiate degrees of adiposity in people without diabetes, highlighting the value of integrated approaches in capturing obesity-related biological burden. These findings support the concept that systemic inflammation may represent a graded and potentially threshold-dependent marker of adiposity, even in the absence of overt metabolic disease, whereas barrier-related pathways may reflect more context-dependent or advanced processes. However, these observations should be interpreted as cross-sectional associations rather than evidence of temporal progression or causality. Future studies should aim to validate these results in larger and more diverse populations, explore longitudinal associations with clinical outcomes, and assess whether such composite indices provide incremental value in risk stratification and therapeutic decision-making. Further refinement of multi-biomarker models, potentially incorporating additional biological domains, may enhance their utility in advancing precision medicine approaches in obesity and metabolic health.

## Figures and Tables

**Table 1:** Mean values of patient characteristics across BMI categories.

	BMI Category (kg/m^2^)			
Parameter	<25 (*n* = 20)	25–29.9 (*n* = 34)	≥30 (*n* = 34)	*p*-Value
Age (years)	43.45 ± 11.98	49.88 ± 13.01	47.44 ± 10.09	0.163
BMI (kg/m^2^)	22.51 ± 1.29	27.25 ± 1.22	34.32 ± 3.83	<0.01
hs-CRP (mg/dL)	0.19 ± 0.28	0.21 ± 0.28	0.35 ± 0.41	0.144
Presepsin (ng/mL)	4.78 ± 3.25	3.50 ± 4.33	8.09 ± 13.79	0.008
Ferritin (ng/mL)	75.7 ± 35.5	95.2 ± 85.5	110.3 ± 85.7	0.199
IL-6 (pg/mL)	3.39 ± 3.84	3.58 ± 3.94	7.50 ± 20.45	0.126
β-defensin-2 (pg/mL)	202.5 ± 180.7	173.9 ± 88.2	164.6 ± 30.0	0.976
REG3α (ng/mL)	521.5 ± 311.3	646.0 ± 217.1	576.3 ± 203.3	0.226

Abbreviations: BMI, body mass index; hs-CRP, high-sensitivity C-reactive protein; IL-6, interleukin-6; REG3α, regenerating islet-derived protein 3 alpha.

**Table 2:** Pairwise comparisons and effect sizes of the Inflammatory Load Index across BMI categories.

Comparison Between BMI Categories (kg/m^2^)	Mean Difference (SD Units)	Cohen’s d	Adjusted *p*-Value
≥30 vs. 25–29.9	0.45	0.80	0.032
≥30 vs. <25	0.31	0.56	0.126
25–29.9 vs. <25	−0.14	0.25	0.373

Positive values indicate higher Inflammatory Load Index in the first group listed. Abbreviations: BMI, body mass index; SD, standard deviation.

**Table 3:** Comparative discriminatory performance of composite indices and individual biomarkers for obesity vs. overweight.

Variable	AUC (95% CI)
Inflammatory Load Index	0.720 (0.576–0.851)
Presepsin	0.679 (0.529–0.807)
hs-CRP	0.619 (0.469–0.756)
Ferritin	0.555 (0.406–0.704)
IL-6	0.510 (0.354–0.666)
Barrier Activation Index	0.418 (0.259–0.576)

Abbreviations: AUC, area under the curve; CI, confidence interval; hs-CRP, high-sensitivity C-reactive protein; IL-6, interleukin-6.

## Data Availability

The data presented in the study are available on request from the corresponding author. The data are not publicly available due to privacy restrictions of the Greek National Health System.
